# Southern Medical College

**Published:** 1888-08

**Authors:** 


SOUTHERN MEDICAL COLLEGE.—See the advertisement of this vigorous young school.

